# Gene and microRNA Expression Profiling in Experimental Colitis and Human Ulcerative Colitis: Effects of *STAT6* Knockout and *Corylus avellana* Treatment

**DOI:** 10.3390/ijms27219411

**Published:** 2026-10-22

**Authors:** Iuliana Samoilă, Cristian George Țieranu, Anca Hermenean, Sorina Dinescu

**Affiliations:** 1 Department of Biochemistry and Molecular Biology, Faculty of Biology, University of Bucharest, 050095 Bucharest, Romania; 2 Department of Gastroenterology, Elias Emergency University Hospital, 011461 Bucharest, Romania; 3 “Aurel Ardelean” Institute of Life Sciences, Vasile Goldis Western University, 310414 Arad, Romania; 4 Research Institute of the University of Bucharest, 050663 Bucharest, Romania

**Keywords:** inflammatory bowel diseases, ulcerative colitis, inflammation, autophagy, endoplasmic reticulum stress, unfolded protein response, miRNAs, *STAT6*

## Abstract

Inflammatory bowel diseases (IBDs) are chronic immune-mediated disorders of the gastrointestinal tract resulting from a complex interplay between genetic susceptibility and dysregulated immune responses. To improve the understanding of the molecular mechanisms’ underlying disease pathogenesis, mouse models with experimental colitis and patients with ulcerative colitis (UC) were investigated for the expression of genes and small non-coding RNAs involved in key pathogenic pathways. In experimental colitis, the potential modulatory effects of *STAT6* knockout and *Corylus avellana* treatment were also evaluated. Our findings revealed significant transcriptional alterations in molecular markers associated with inflammation, epithelial barrier integrity, autophagy, and endoplasmic reticulum stress, highlighting their association with the development and persistence of chronic intestinal inflammation. Both *STAT6* deletion and *Corylus avellana* extract treatment modulated several of these alterations, with *STAT6* knockout showing a more pronounced effect on the molecular profile associated with experimental colitis. In addition, miRNA expression profiling revealed a panel of dysregulated miRNAs, while bioinformatic analysis identified potential miRNA-gene regulatory interactions, supporting their possible involvement in the post-transcriptional regulation of these biological processes. Overall, the combined analysis of gene and miRNA expression profiles highlights molecular alterations associated with experimental colitis and human UC and suggests potential regulatory interactions that require further experimental validation.

## 1. Introduction

IBDs, including Crohn’s disease and UC, are chronic inflammatory disorders of the gastrointestinal tract that arise from the disruption of intestinal homeostasis, resulting from a complex interplay between genetic susceptibility, environmental factors, an aberrant immune response, and alterations of the intestinal microbiota [1,2,3,4].

The intestinal epithelium represents the first line of defense against luminal microorganisms, and alteration of the mucus layer and intercellular junctions, including occludin (OCLN) and claudins (CLDN), compromises the epithelial barrier integrity. This allows for bacterial translocation and promotes excessive activation of immune responses, characterized by the production of large amounts of pro-inflammatory cytokines, such as IL-6 and TNF-α, which further perpetuate a vicious cycle of chronic inflammation [5,6,7].

Besides barrier dysfunction and inflammation, increasing evidence highlights defective autophagy and endoplasmic reticulum stress (ERS) as key contributors to UC pathogenesis. Chronic intestinal inflammation can modify the expression of autophagy-related genes, including *ATG16L1*, *MAP1LC3B,* and *SQSTM1* [8], as well as induce ERS, succeeded by unfolded protein response (UPR) activation, primarily through the PERK-, IRE1/XBP1-, and ATF6-signaling pathways. Imbalanced autophagy and prolonged ERS can contribute to impaired bacterial clearance, sustained inflammatory signaling, and apoptosis [9,10].

Post-transcriptional regulation mediated by microRNAs (miRNAs/miR) has emerged as an important mechanism controlling intestinal inflammation, and many miRNAs have been reported to regulate a number of cellular processes associated with UC. For example, miR-21 and miR-155 are major regulators of pro-inflammatory cytokine production [11], whereas miR-146 family members modulate NF-kB activation, autophagic flux, and epithelial barrier integrity [12].

The complexity of the molecular mechanisms underlying UC has also led to increasing interest in therapeutic strategies targeting specific signaling pathways, as well as in complementary approaches based on naturally derived bioactive compounds. Among the molecular pathways potentially relevant to UC, STAT6 is a major downstream mediator of IL-4 and IL-13 signaling and is involved in the regulation of intestinal immune responses and epithelial homeostasis. IL-13/STAT6 signaling can influence epithelial permeability, cell turnover, and barrier integrity, processes that are highly relevant to intestinal inflammation [13,14]. Therefore, genetic deletion of *STAT6* provides a useful mechanistic approach for investigating how the absence of *STAT6*-dependent signaling influences the development and severity of experimental colitis. In parallel, naturally derived compounds have attracted interest as potential adjuvant strategies due to their anti-inflammatory, antioxidant, and tissue-protective properties. *Corylus avellana* extract represents a potential source of bioactive compounds with relevant biological activities, such as phenolic acids (gallic acid) and flavonoids (quercitin, naringenin), supporting the investigation of its effects as an adjuvant intervention in experimental colitis [15].

Given the complex crosstalk among inflammation, epithelial barrier dysfunction, autophagy, and ERS, an integrated molecular approach is essential for a comprehensive understanding of UC pathogenesis. Therefore, the present study aimed to investigate the molecular mechanisms underlying experimental colitis in mice and ulcerative colitis in humans by evaluating the expression of key genes and miRNAs involved in these interconnected biological processes. Furthermore, the effects of *STAT6* deletion and *C. avellana* treatment were assessed in a murine model of experimental colitis to evaluate their impact on the disease progression and their potential protective mechanisms.

## 2. Results

### 2.1. Molecular Expression Profiling in Murine TNBS-Induced Experimental Colitis

#### 2.1.1. Gene Expression Profiling

To investigate the molecular effects of *STAT6* deletion and *Corylus avellana* treatment in experimental colitis, the relative mRNA expression of genes involved in epithelial barrier integrity, inflammation, autophagy, and UPR was evaluated by RT-qPCR in all experimental groups.

**Figure 1:**
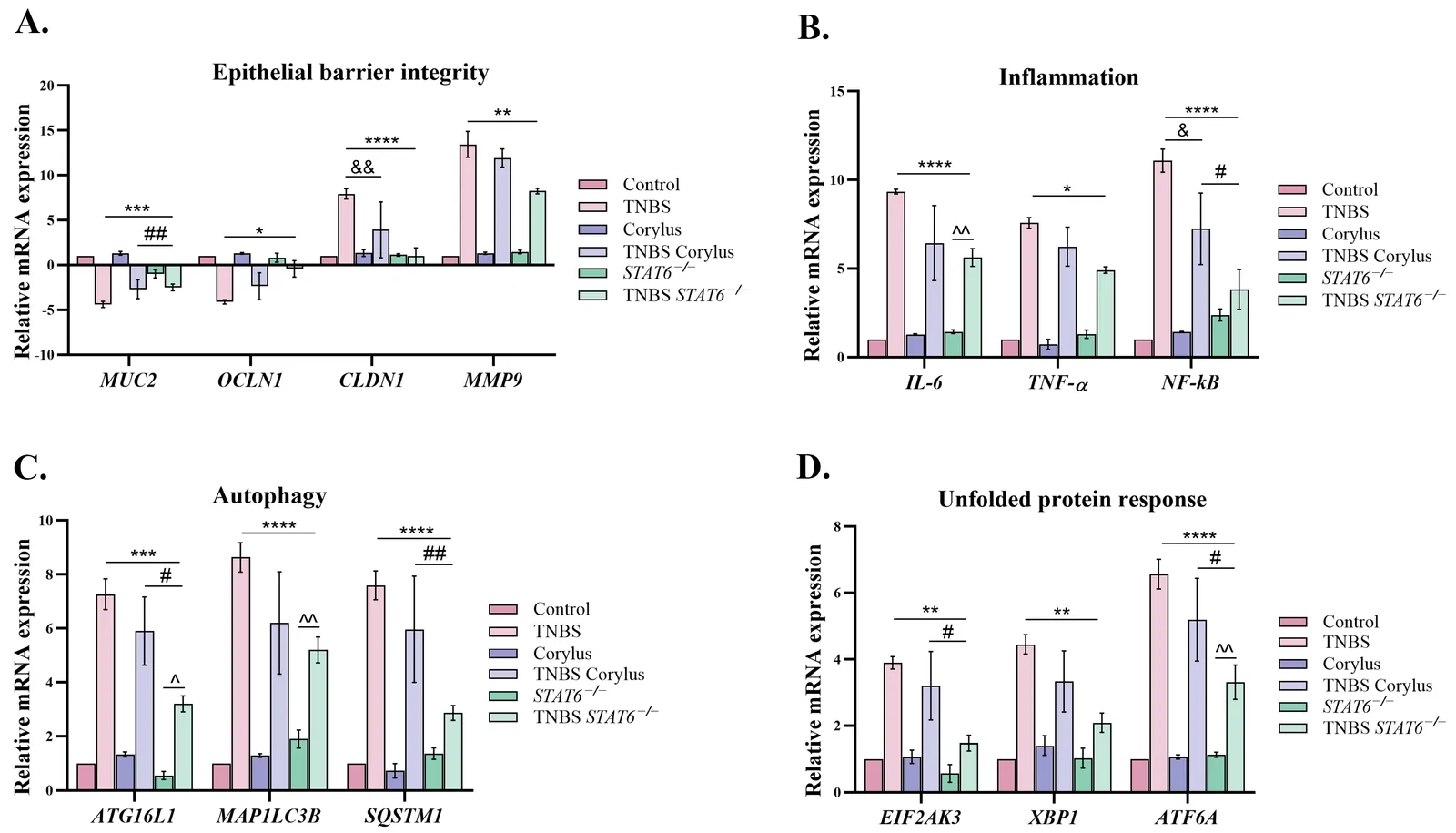
Relative mRNA expression of genes associated with autophagy, UPR, epithelial barrier integrity, and inflammation in mouse models. Relative mRNA levels of (**A**) epithelial barrier-related genes, together with MMP9, (**B**) inflammatory mediators, (**C**) autophagy-related genes, and (**D**) UPR-associated markers were quantified by RT-qPCR. Statistical significance is indicated as follows: “*” symbolizes the comparison between TNBS and TNBS *STAT6*^−/−^ conditions, where * indicates *p* < 0.05, ** *p* < 0.01, *** *p* < 0.001, and **** *p* < 0.0001, respectively; “&” symbolizes the comparison between TNBS and TNBS Corylus conditions, where & means *p* < 0.05 and && means *p* < 0.01; “#” symbolizes the comparison between TNBS Corylus and TNBS *STAT6*^−/−^ conditions, where # accounts for *p* < 0.05 and ## for *p* < 0.01; “^” symbolizes the comparison between *STAT6*^−/−^ and TNBS *STAT6*^−/−^ conditions, where ^ indicates *p* < 0.05 and ^^ *p* < 0.01.

Compared with the Control group, experimental colitis mice showed decreased *MUC2* expression, whereas TNBS *STAT6*^−/−^ mice displayed significantly higher expression than the TNBS group (*p* < 0.001). *MUC2* expression was also higher in the TNBS Corylus group (*p* < 0.01) than in TNBS *STAT6*^−/−^ mice, while no significant difference was observed between the TNBS and TNBS Corylus groups (Figure 1A). *OCLN1* expression was decreased in experimental colitis compared with Control but increased in TNBS *STAT6*^−/−^ mice (*p* < 0.05) relative to the experimental colitis group. No significant difference was detected between the TNBS *STAT6*^−/−^ and TNBS Corylus groups. On the other hand, *CLDN1* expression was increased following experimental colitis induction compared with controls. In contrast, *CLDN1* expression was increased in experimental colitis but significantly attenuated by *STAT6* deletion (*p* < 0.0001) and *Corylus avellana* treatment (*p* < 0.01). The increased expression of *MMP9* was attenuated in both intervention groups, with the lowest expression with statistical differences observed in TNBS *STAT6*^−/−^ mice (*p* < 0.01), but no statistical difference was reached for TNBS Corylus.

In regard to the inflammatory response associated with experimental colitis, *IL-6* displayed an increased expression in experimental colitis mice compared with the Control group (Figure 1B). However, *STAT6* deletion in TNBS mice resulted in lower *IL-6* expression relative to the TNBS group (*p* < 0.0001), but in higher expression (*p* < 0.01) when compared to the *STAT6*^−/−^ group. Although *IL-6* expression also tended to decrease in the TNBS Corylus group compared to experimental colitis mice, the difference was not statistically significant. A similar pattern was observed for *TNF-α*, which was increased in experimental colitis mice, showing higher values than the Control, whereas TNBS *STAT6*^−/−^ mice exhibited significantly reduced expression compared with the TNBS group (*p* < 0.05). *NF-kB* expression was approximately 11-fold higher in experimental colitis than in the Control group, with the TNBS group having a strong increase in expression (*p* < 0.0001) when compared to TNBS *STAT6*^−/−^ mice. *STAT6* deletion significantly reduced *NF-kB* expression in TNBS mice when compared to TNBS Corylus (*p* < 0.05), but TNBS Corylus registered lower expression compared to the TNBS group (*p* < 0.05).

Autophagy-related genes were upregulated in TNBS mice compared with the Control, namely *ATG16L1* (~7.5-fold change), *MAP1LC3B* (~9-fold change), and *SQSTM1* (~8-fold change). *ATG16L1* expression was significantly lower in TNBS *STAT6*^−/−^ mice than in the experimental colitis group (*p* < 0.001) but remained higher than in the *STAT6*^−/−^ group (*p* < 0.05). Similarly, *MAP1LC3B* expression was higher in TNBS *STAT6*^−/−^ than in *STAT6*^−/−^ mice (*p* < 0.01). *STAT6* knockout in TNBS-treated mice also attenuated *SQSTM1* expression, showing lower values than the TNBS group (*p* < 0.0001) and the TNBS Corylus group (*p* < 0.01). Although the *ATG16L1*, *MAP1LC3B,* and *SQSTM1* expressions were lower in TNBS Corylus than in TNBS mice, these differences were not statistically significant (Figure 1C).

Altered expression was also identified for UPR-associated genes, with *EIF2AK3*, *XBP1*, and *ATF6A* showing increased transcript levels in TNBS mice compared with the Control group. In TNBS *STAT6*^−/−^ mice, *EIF2AK3* and XBP1 expression was significantly reduced (*p* < 0.01) compared with the TNBS group (Figure 1D). ATF6A expression was also significantly lower in TNBS *STAT6*^−/−^ mice than in both TNBS (*p* < 0.0001) and TNBS Corylus mice (*p* < 0.05) but remained higher than in *STAT6*^−/−^ mice (*p* < 0.01). In contrast, no significant changes were observed in *EIF2AK3* and *XBP1* expression for the TNBS Corylus group compared with the experimental colitis, indicating a more consistent effect of *STAT6* deletion than of *Corylus avellana* treatment.

#### 2.1.2. Differential miRNA Expression and Functional Analysis

**Figure 2:**
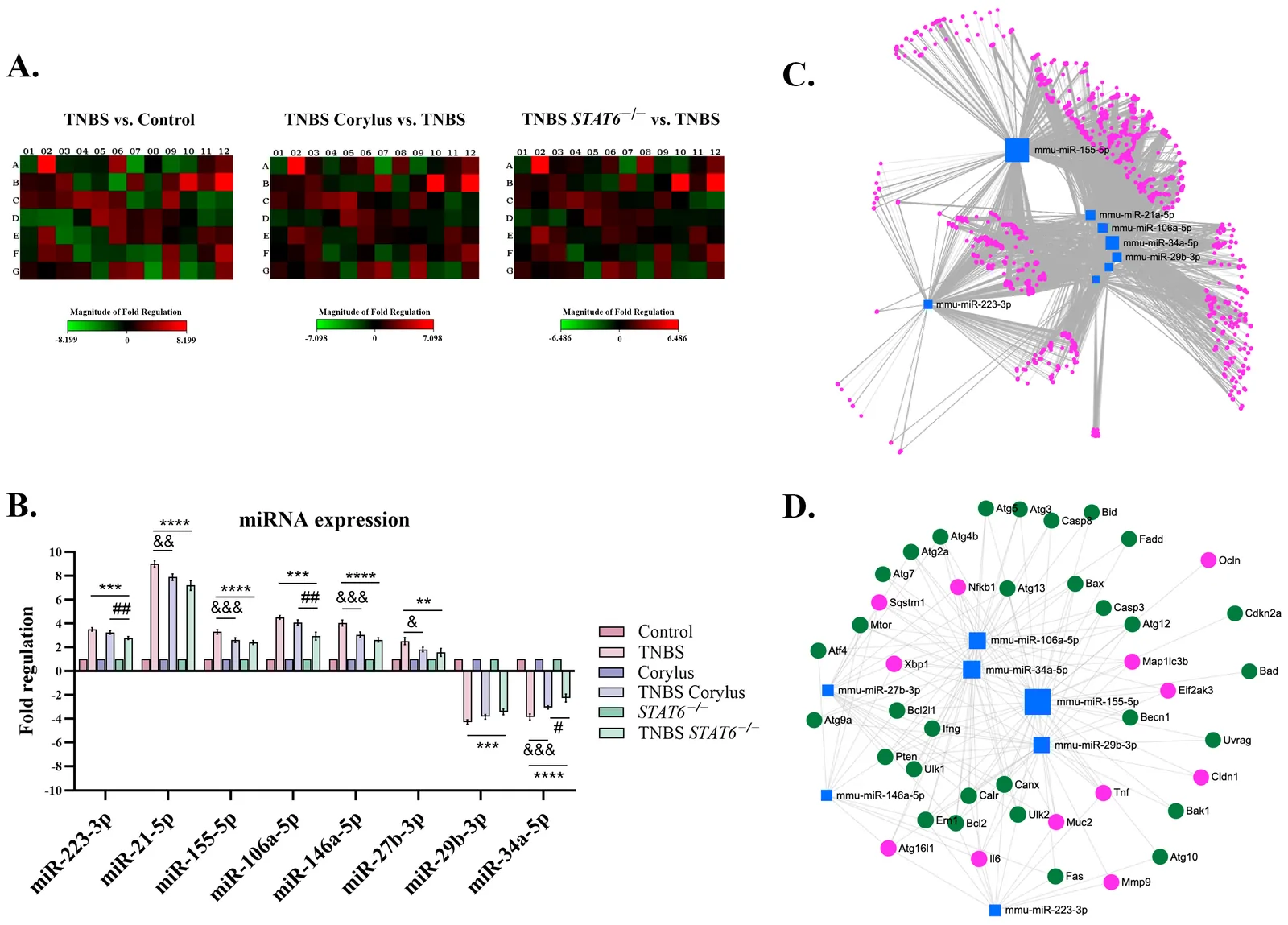
Integrated analysis of miRNA expression and miRNA-target interaction networks in experimental colitis. (**A**) Heatmaps showing differential miRNA expression between experimental colitis vs. Control, TNBS Corylus vs. TNBS, and TNBS *STAT6*^−/−^ vs. TNBS. Rows (A–G) and columns (01–12) correspond to the miRNA positions on the qPCR array plate (detailed in Supplementary Material Table S1A). (**B**) Relative expression of selected miRNAs. (**C**) Global miRNA-target interaction network generated using miRNet 2.0, based on validated interactions obtained from miRTarBase v9.0 and TarBase v9.0, comprising 14,722 target genes associated with the selected dysregulated miRNAs (backbone layout). (**D**) Focused miRNA-gene interaction network displaying potential regulatory relationships between the selected miRNAs and target genes related to inflammation, epithelial barrier function, autophagy, ERS, and apoptosis. Blue squares represent miRNAs, pink circles indicate genes investigated in the present study, and green circles represent additional genes associated with the biological processes of interest. Statistical significance is indicated as follows: “*” symbolizes the comparison between TNBS and TNBS *STAT6*^−/−^ conditions, where ** indicate *p* < 0.01, *** *p* < 0.001, and **** *p* < 0.0001, respectively; “&” symbolizes the comparison between TNBS and TNBS Corylus conditions, where & means *p* < 0.05, && means *p* < 0.01 and &&& means *p* < 0.001; “#” symbolizes the comparison between TNBS Corylus and TNBS *STAT6*^−/−^ conditions, where # accounts for *p* < 0.05 and ## for *p* < 0.01.

Comparative analysis of the miRNA expression profile revealed a distinct molecular pattern involved in experimental colitis and its modulation following *STAT6* deletion or *Corylus avellana* treatment. Heatmap visualization displayed several dysregulated colitis-associated miRNAs in the TNBS-treated group compared with the Control group, whereas both TNBS *STAT6*^−/−^ and TNBS Corylus mice showed a partially restored expression profile toward physiological levels (Figure 2A; for additional information regarding analyzed miRNAs, please see Table S1A–E in Supplementary Material). Among the 84 miRNAs evaluated, eight miRNAs were selected for further analysis based on their previously reported relevance to UC and related molecular mechanisms, as well as their different expression patterns across the experimental groups. These miRNAs were then used for the miRNA–target interaction analyses.

Quantitative analysis of the selected miRNAs revealed that miR-223-3p, miR-21-5p, miR-155-5p, miR-106a-5p, miR-146a-5p, and miR-27b-3p were upregulated, whereas miR-29b-3p and miR-34a-5p were downregulated in experimental colitis relative to the Control (Figure 2B). Compared with the TNBS-treated group, *STAT6* deletion significantly reduced the expression of miR-21-5p, miR-155-5p, miR-146a-5p (*p* < 0.0001), miR-223-3p, and miR-106a-5p (*p* < 0.001), as welll as of miR-27b-3p (*p* < 0.05). Moreover, it significantly attenuated the downregulation of miR-34a-5p (*p* < 0.0001) and miR-29b-3p (*p* < 0.01). Similarly, *Corylus avellana* treatment significantly decreased the expression of miR-155-5p, miR-146a-5p, and miR-34a-5p (*p* < 0.001), together with miR-21-5p (*p* < 0.01), compared with the experimental colitis group. Comparison between the two interventions further revealed a significantly stronger effect of *STAT6* deletion on the normalization of miR-223-3p and miR-106a-5p (*p* < 0.01), but also for miR-34a-5p (*p* < 0.05), whereas no significant differences were observed for the remaining miRNAs. These findings indicate that both interventions could modulate the TNBS-induced colitis-associated miRNA dysregulation, with *STAT6* deletion exerting the more pronounced modulatory effect.

To further investigate the biological significance of the selected dysregulated miRNAs, a global miRNA-target interaction network was generated using miRNet 2.0 platform (Figure 2C), where 14,722 target genes were identified. To integrate the miRNA analysis with the experimentally evaluated transcriptional alterations, a focused miRNA-gene interaction network was subsequently generated, to visualize potential regulatory relationships relevant to the molecular processes evaluated in this study (Figure 2D). From the miRNA-associated targets identified in the global network, genes involved in the major biological processes investigated were selected, including inflammation (*IL6*, *TNF*, *NFKB1*), epithelial barrier integrity (*MUC2*, *OCLN*, *CLDN1*), autophagy (*ATG16L1*, *MAP1LC3B*, *SQSTM1*), and UPR (*EIF2AK3*, *XBP1*).

Functional enrichment analysis of the miRNA-associated target genes was performed using the Kyoto Encyclopedia of Genes and Genomes (KEGG) database. The analysis identified multiple significantly enriched pathways, among which six significant ones were selected for presentation based on their direct relevance to the biological processes investigated in this study (Table 1).

### 2.2. Molecular Expression Profiling in Human UC

Following the characterization of molecular alterations in experimental colitis and their modulation by *STAT6* deletion and *Corylus avellana* treatment, we next investigated whether alterations involving the same major biological processes were also identified in human UC. Therefore, mucosal colonic biopsies from healthy individuals and patients with UC were subjected to gene and miRNA expression profiling using qPCR arrays in order to identify molecular modifications related to intestinal inflammation and disease pathogenesis.

#### 2.2.1. Gene Expression Profiling

To obtain an overview of the molecular alterations associated with UC, the expression of a broad panel of genes involved in autophagy, UPR, and related cellular processes was assessed.

**Figure 3:**
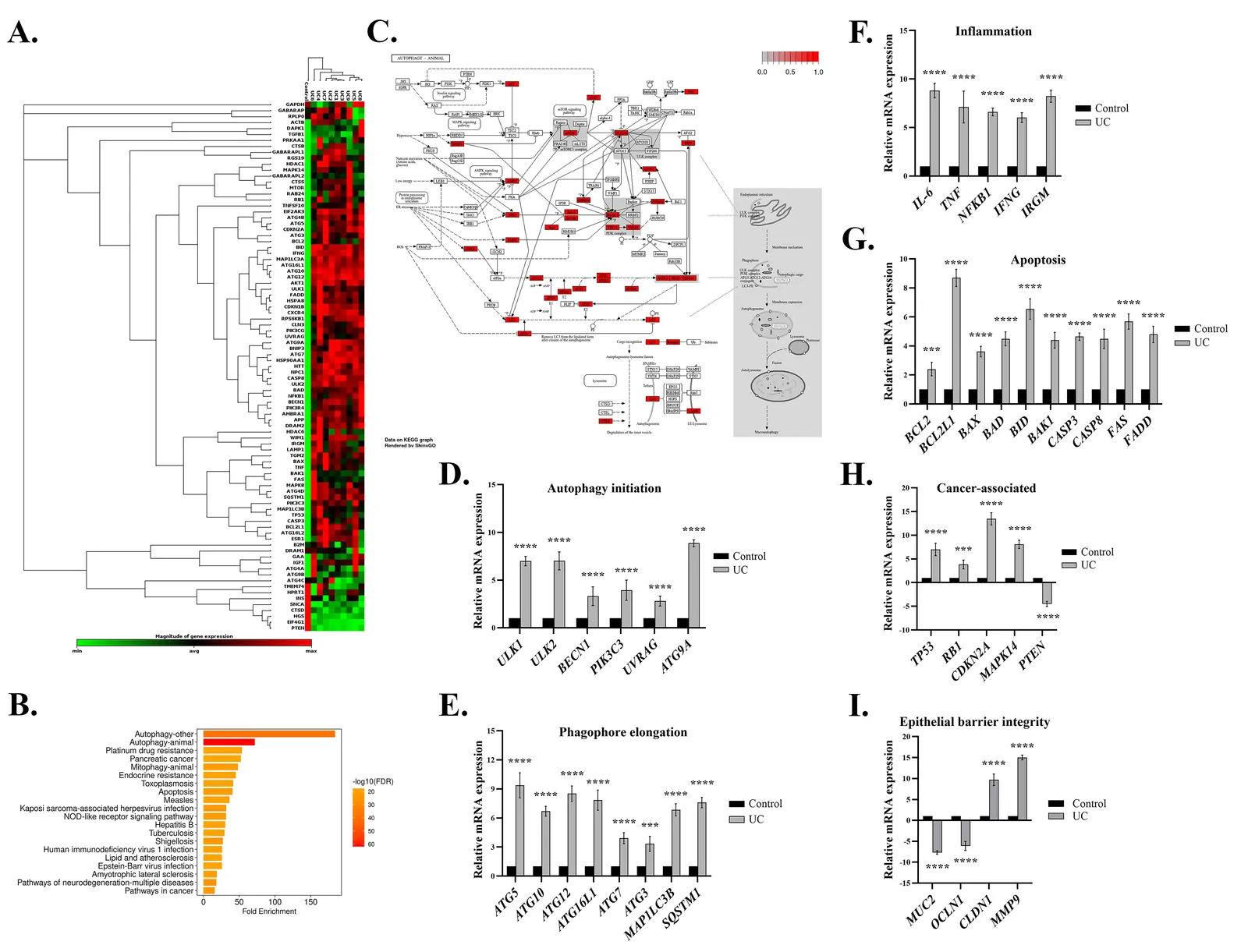
Differential expression of genes evaluated with the PCR Array Human Autophagy plates in colonic biopsies from healthy individuals (Control) and patients with ulcerative colitis (UC). (**A**) Hierarchical clustering heatmap illustrating the overall expression profile of the analyzed genes. Green indicates low expression, black indicates intermediate expression, and red indicates high expression; (**B**) Bar plot showing enriched KEGG pathways of autophagy-associated genes, with bar length representing fold enrichment and color indicating statistical significance based on −log10(FDR). Red bars indicate higher statistical significance, whereas orange bars indicate lower statistical significance (plot theme set to “linedraw”); (**C**) KEGG autophagy pathway map generated using ShinyGO version 0.86, highlighting genes from the input gene list mapped to the pathway; (**D**) Relative mRNA expression of genes involved in autophagy initiation; (**E**) Relative mRNA expression of genes associated with phagophore elongation; (**F**) Relative mRNA expression of inflammatory markers; (**G**) Relative mRNA expression of apoptosis-related genes, both intrinsic and extrinsic pathways, including anti-apoptotic and pro-apoptotic markers; (**H**) Relative mRNA expression of cancer-associated genes; (**I**) Relative mRNA expression of epithelial barrier-related genes. Statistical significance: *** *p* < 0.001; **** *p* < 0.0001.

Hierarchical clustering analysis revealed distinct transcriptional profiles between healthy controls and UC patients (Figure 3A; for additional information please see Table S1F in Supplementary Material). The heatmap indicated widespread transcriptional alterations in genes associated with intestinal homeostasis. KEGG pathway enrichment analysis of the upregulated genes revealed a marked enrichment of autophagy-related pathways, particularly Autophagy–other and Autophagy–animal, alongside pathways associated with mitophagy, apoptosis, and NOD-like receptor signaling (Figure 3B). Mapping of the upregulated genes onto the KEGG Autophagy–animal pathway further illustrated their distribution across several processes involved in autophagy regulation, including upstream signaling, autophagosome initiation, and formation and lysosomal degradation (Figure 3C). In addition, several genes associated with autophagy regulation, apoptosis, and cellular stress showed increased expression in UC samples. To further characterize these transcriptional alterations, representative genes associated with autophagy, inflammation, apoptosis, cancer-related pathways, and epithelial barrier integrity were analyzed individually.

Genes involved in the initiation of autophagy were differentially expressed between healthy controls and patients with UC (Figure 3D). Compared with the Control group, UC biopsies exhibited significantly increased expression of *ULK1* and *ULK2* (*p* < 0.0001). Similarly, *BECN1* and *PIK3C3* (*p* < 0.0001), two key regulators of autophagosome initiation, were increased in UC An approximately three times upregulation of *UVRAG* (*p* < 0.0001) was also observed in the UC group. In addition, *ATG9A*, another gene involved in the early stages of autophagy, showed significantly higher expression in UC than in the Control (*p* < 0.0001).

Elongation and maturation phases of autophagy also exhibited altered transcriptional expression of key genes in UC biopsies compared with healthy controls (Figure 3E). Significant upregulation (*p* < 0.0001) of *ATG7* (4-fold regulation), *ATG10* (7-fold regulation), and *ATG16L1* (8-fold regulation), along with approximately 9-times higher expression of *ATG12,* was observed in the UC group. *ATG3* and *ATG5* (*p* < 0.001 and *p* < 0.0001, respectively) were significantly upregulated in UC samples compared with Control, as well as *MAP1LC3B* and *SQSTM1* (*p* < 0.0001).

Significant overexpression of inflammatory markers (*p* < 0.0001) in mucosal colonic biopsies from UC patients when compared to healthy controls, specifically *IL-6*, *TNF*, *NFKB1*, *IFNG,* and *IRGM*, is highlighted in Figure 3F. Among these, the highest fold regulations were observed for *IL-6*, with a 9-fold difference, *NFKB1* and *IRGM*, which showed an approximately 8-fold regulation, all indicating a pronounced inflammatory transcriptional profile in the colonic mucosa of UC patients and an activation of multiple inflammatory pathways.

The anti-apoptotic *BCL2* gene exhibited a 2-fold increase in expression (*p* < 0.001) in UC when compared with the Control group (Figure 3G), whereas *BCL2L1* was approximately 9 times higher in the UC group (*p* < 0.0001). Moreover, the pro-apoptotic marker *BAX* was upregulated in UC samples (*p* < 0.0001), showing an approximately 4-fold increase in expression. Similarly, *BAD*, *BID*, *BAK1,* and *CASP3*, all part of the intrinsic apoptotic pathway, exhibited significantly higher expression (*p* < 0.0001) in the UC group compared to the Control group. The extrinsic apoptotic pathway members, *CASP8*, *FAS,* and *FADD* (*p* < 0.0001), also showed an upregulation in their expression in the UC colonic biopsies.

*TP53* gene expression was significantly upregulated in the UC group (*p* < 0.0001), indicating substantial transcriptional alteration of this major tumor-suppressor pathway. In addition, *RB1* (*p* < 0.001) and *MAPK14* (*p* < 0.0001) showed higher expression levels in UC biopsies than in control samples. *CDKN2A* showed a pronounced change in its expression (Figure 3H), with an approximately 14-fold increase in the UC group (*p* < 0.0001). In contrast, *PTEN* was significantly downregulated in UC biopsies compared with healthy controls (*p* < 0.0001).

Analysis of epithelial barrier-related genes revealed a distinct expression pattern in UC biopsies compared with healthy controls (Figure 3I). The mucin-encoding gene *MUC2* was significantly downregulated in the UC group (*p* < 0.0001), indicating reduced expression of a major component of the intestinal mucus layer. In the same way, expression of the tight junction protein *OCLN1* was decreased in UC biopsies (*p* < 0.0001). In contrast, *CLDN1* displayed an approximately 10-fold increase in expression (*p* < 0.0001), while *MMP9* had an approximately 15-fold higher expression in the UC group (*p* < 0.0001).

**Figure 4:**
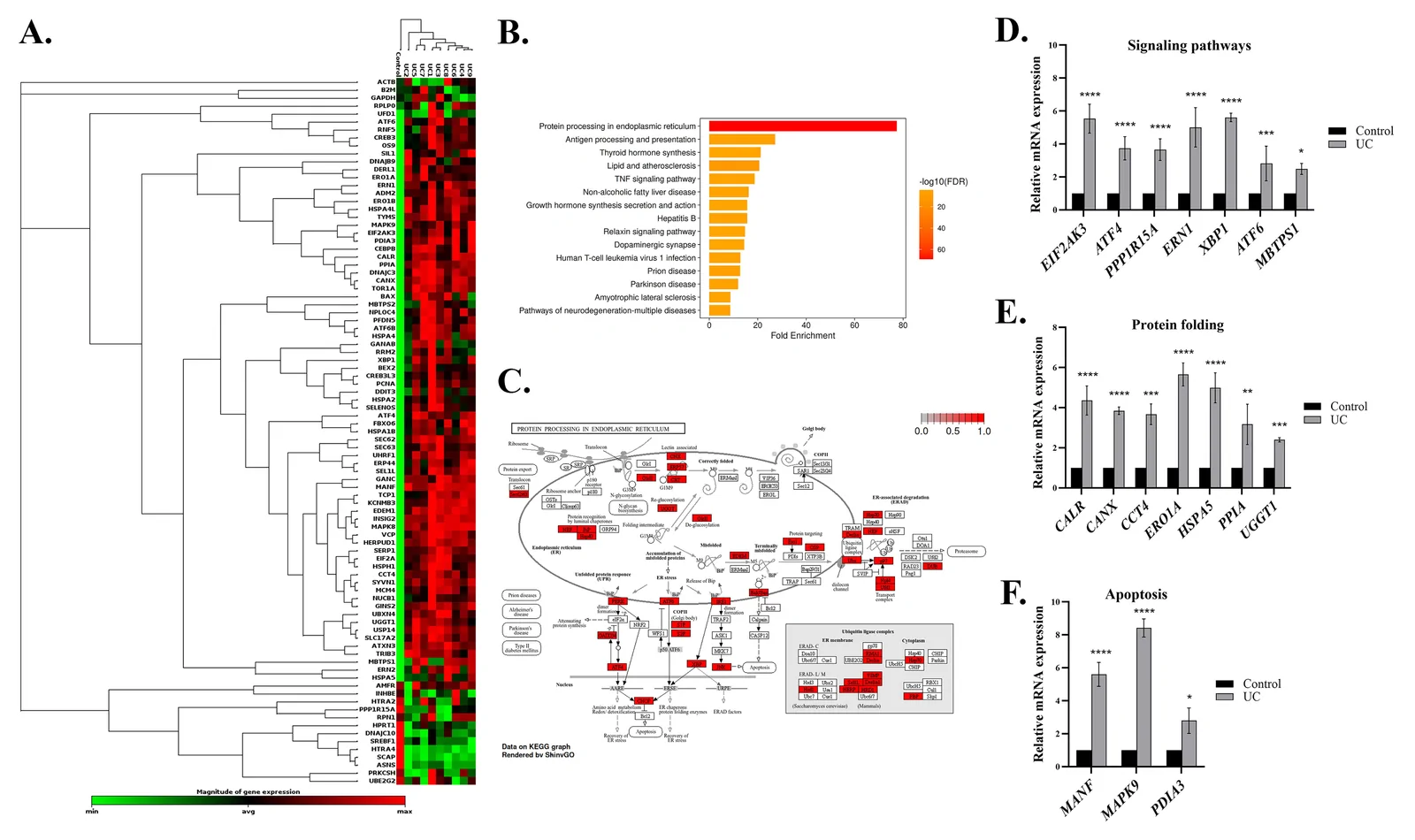
Differential expression of genes evaluated with the PCR Array Human UPR plates in colonic biopsies from healthy individuals (Control) and patients with ulcerative colitis (UC). (**A**) Hierarchical clustering heatmap illustrating the expression profiles of the analyzed genes. Green indicates low expression, black indicates intermediate expression, and red indicates high expression; (**B**) Bar plot showing enriched KEGG pathways of UPR-associated genes, with bar length representing fold enrichment and color indicating statistical significance based on −log10(FDR). Red bars indicate higher statistical significance, whereas orange bars indicate lower statistical significance (plot theme set to “linedraw”); (**C**) KEGG UPR pathway map generated using ShinyGO, highlighting genes from the input gene list mapped to the pathway; (**D**) Relative mRNA expression of genes involved in UPR-signaling pathways; (**E**) Relative mRNA expression of genes associated with protein folding; (**F**) Relative mRNA expression of apoptosis-related gene markers. Statistical significance: * *p* < 0.05; ** *p* < 0.01; *** *p* < 0.001; **** *p* < 0.0001.

Hierarchical clustering analysis of UPR-related genes demonstrated a clear separation between healthy controls and UC samples, reflecting distinct transcriptional signatures associated with endoplasmic reticulum stress (Figure 4A; for additional information, please see Table S1G in Supplementary Material). To further characterize the functional relevance of the differentially expressed genes, KEGG pathway enrichment analysis was performed. Among the upregulated genes, Protein processing in the endoplasmic reticulum was the most prominently enriched pathway, followed by antigen processing and presentation and other inflammation-related pathways, including TNF signaling (Figure 4B). Mapping of the upregulated genes onto the KEGG protein processing pathway further highlighted their involvement in protein folding, UPR signaling, and ER-associated degradation (Figure 4C). Several genes involved in UPR signaling, molecular chaperone activity, and apoptosis displayed altered expression in UC biopsies, indicating transcriptional alterations in ER stress-related processes. Representative genes from these functional categories were subsequently selected for individual expression analysis.

To characterize transcriptional alterations related to the three major UPR signaling branches, representative genes belonging to the PERK, IRE1, and ATF6 pathways were analyzed. Genes represented in Figure 4D exhibited significantly higher expression in UC biopsies compared with healthy controls. Within the PERK pathway, *EIF2AK3* (*p* < 0.0001) showed the highest expression (~5.5-fold increase), followed by *ATF4* and *PPP1R15A* (*p* < 0.0001), both displaying approximately 3.5-fold upregulation. Similarly, the IRE1-associated genes *ERN1* and *XBP1* (*p* < 0.0001) showed significantly increased expression in the UC compared to the Control group, with approximately 5.5-fold and 6-fold increases. Moreover, genes associated with the ATF6 branch were also significantly altered, although to a lesser extent, with *ATF6* and *MBTPS1* showing approximately 2.8-fold (*p* < 0.001) and 2.5-fold (*p* < 0.05) increases, respectively.

A significantly higher expression in UC biopsies compared to the Control group was observed for *CALR, CANX,* and *HSPA5* (*p* < 0.0001), along with *ERO1A*, which displayed the highest expression levels, reaching an approximately 5.6-fold increase (*p* < 0.0001). *CCT4* was also significantly upregulated (*p* < 0.001) in the UC group (Figure 4E), whereas *PPIA* and *UGGT1* displayed a lower, yet statistically significant, increase (*p* < 0.01 and *p* < 0.001, respectively).

Among the apoptosis-related genes evaluated in the UPR context, *MAPK9* exhibited the highest expression (Figure 4F), showing an approximately 8.4-fold increase compared with healthy controls (*p* < 0.0001). *MANF* was also upregulated approximately 5.5 times (*p* < 0.0001), whereas *PDIA3* displayed a more moderate but significant increased expression of approximately 2.8-fold (*p* < 0.05). The coordinated upregulation of these ERS-associated genes indicates an increased transcriptional demand for protein folding and quality-control mechanisms in the inflamed colonic mucosa of patients with UC.

#### 2.2.2. Differential miRNA Expression

**Figure 5:**
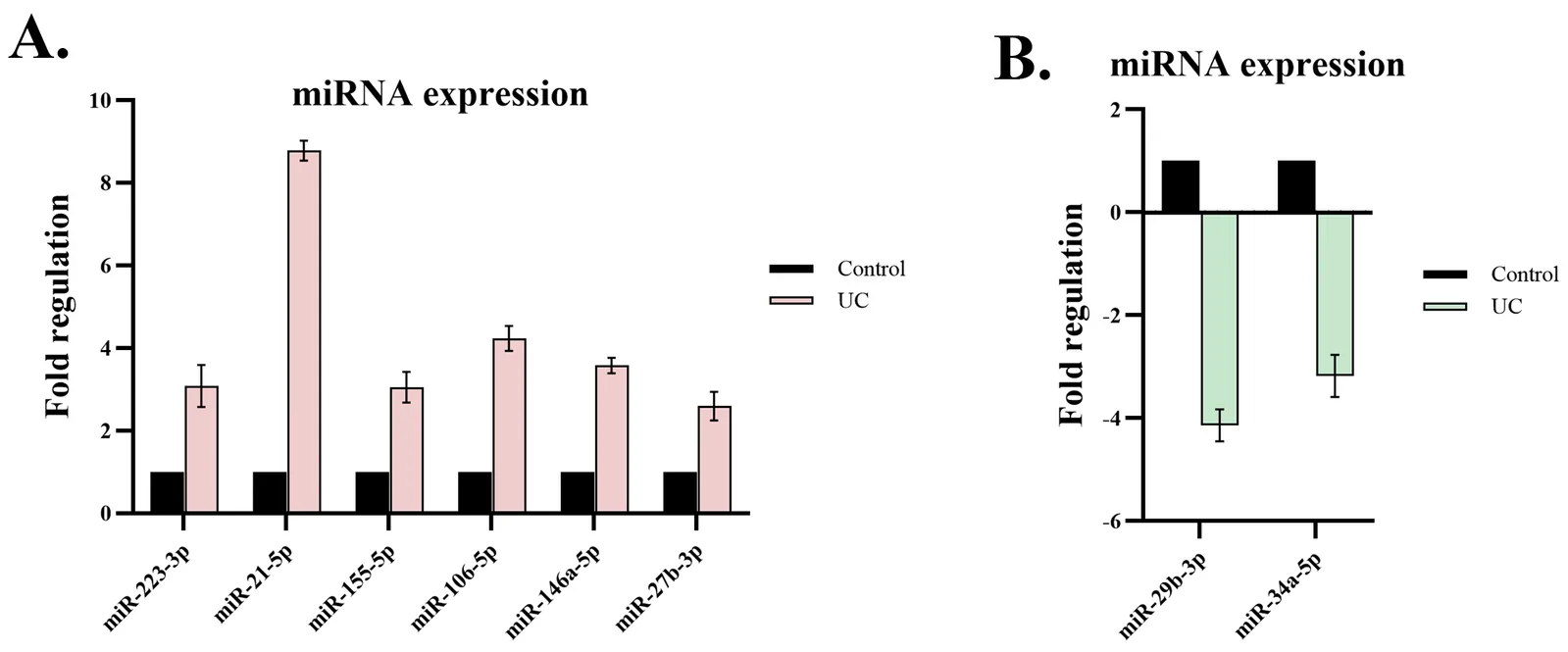
Differential miRNA expression profile in UC. (**A**) Relative expression of upregulated miRNAs and (**B**) downregulated miRNAs in the UC group compared with controls. miRNA expression was quantified by RT-qPCR.

To validate the relevance of the miRNAs identified in the mice models, their expression was subsequently evaluated in the mucosal colonic biopsies obtained from healthy individuals and patients with UC by RT-qPCR. The selected miRNAs are known to regulate key biological processes involved in UC pathogenesis, including inflammatory signaling, epithelial barrier integrity, autophagy, and immune homeostasis. Among the analyzed miRNAs, miR-21-5p, a well-established regulator of intestinal inflammation and epithelial barrier function, exhibited the highest level of upregulation, showing an approximately 9-fold increase in UC patients compared with healthy controls (Figure 5A). Increased expression was also observed for miR-106a-5p (~4-fold), miR-146a-5p (~3.5-fold), miR-223-3p (~3-fold), miR-155-5p (~3-fold), and miR-27b-3p (~2.5-fold), all of which have been implicated in the regulation of inflammatory and immune responses. In contrast, two miRNAs involved in epithelial homeostasis and cell proliferation, miR-29b-3p and miR-34a-5p, were downregulated in UC biopsies (Figure 5B). miR-29b-3p expression decreased by approximately 4-fold relative to the Control group, whereas miR-34a-5p showed an approximately 3-fold reduction.

## 3. Discussion

IBDs are complex immune-mediated disorders whose pathogenesis is not yet fully understood [16]. Despite considerable progress in identifying their genetic and molecular determinants, the mechanisms responsible for the initiation and persistence of chronic intestinal inflammation remain unclear [17]. Increasing evidence indicates that intestinal homeostasis is maintained through a tightly regulated interplay among several interconnected pathways, whereas their disruption contributes to disease development and progression [18]. Gene and miRNA expression profiling provides a valuable approach for investigating the molecular networks involved in IBD pathogenesis, improving our understanding of disease mechanisms and supporting the identification of potential novel biomarkers and therapeutic targets. Therefore, the present study aimed to evaluate gene and miRNA expression profiles in experimental colitis and patients with UC to investigate molecular alterations associated with inflammation, epithelial barrier dysfunction, autophagy, and ERS, thereby contributing to a better understanding of IBD pathogenesis.

Gene expression profiling in experimental colitis and human UC biopsies identified alterations in the transcriptional landscape associated with disease. While the overall expression patterns differed between mice and patients, both datasets consistently demonstrated dysregulation of genes involved in epithelial barrier integrity, inflammatory signaling, autophagy, and ERS. These findings suggest that UC pathogenesis is driven by the coordinated dysregulation of multiple interconnected biological processes rather than a single molecular pathway.

The intestinal epithelial barrier constitutes the first line of defense against luminal microorganisms, preserving the mucus layer, tight junction proteins, and extracellular matrix remodeling, contributing to intestinal homeostasis. Both experimental colitis and human UC showed a common pattern of epithelial barrier dysfunction, characterized by reduced *MUC2* and *OCLN1* expression and increased *CLDN1* and *MMP9* expression. MUC2, the major gel-forming mucin produced by goblet cells, plays a crucial role in preventing bacterial contact with the intestinal epithelium [19]. Accordingly, *MUC2*-deficient mice exhibit bacterial infiltration into the colonic crypts and spontaneously develop colitis [20]. Likewise, reduced *OCLN1* expression has also been previously reported in patients with UC [21], whereas *CLDN1* overexpression has been suggested as a potential early biomarker of the progression from UC to colitis-associated colorectal cancer (CAC) [22]. Increased *MMP9* expression may further contribute to barrier dysfunction through extracellular matrix degradation and impaired goblet cell differentiation and *MUC2* expression [23]. Interestingly, *STAT6* deletion partially restored the expression of these epithelial barrier markers in our experimental colitis. Similar results were obtained in a previous study on an oxazolone-induced colitis model, where experimental STAT6 deficiency has been shown to attenuate the disease and reduce claudin-2 induction, supporting the involvement of STAT6-dependent signaling in intestinal inflammation and epithelial barrier dysfunction [24]. However, Cosin-Roger et al. reported delayed mucosal healing in *STAT6*^−/−^ mice following TNBS-induced colitis, with impaired M2 macrophage polarization and Wnt signaling [25], suggesting that *STAT6* may have different context-dependent effects on mucosal repair. In the present study, *STAT6* deletion modulated the expression of several genes involved in epithelial barrier integrity, whereas *Corylus avellana* treatment showed a more limited modulatory effect.

Disruption of the mucus barrier facilitates direct contact between luminal bacteria and intestinal epithelial cells, promoting inflammatory responses [20]. In our study, we found an increased expression for *IL-6* and *TNF-*α, which can contribute to macrophage activation and amplification of intestinal inflammation [26]. Moreover, sustained TNF-α signaling may further impair the epithelial barrier by promoting mucus barrier disruption and activation of the NF-κB pathway, amplifying the pro-inflammatory cytokine cascade [27]. The reduced inflammatory marker expression observed under *STAT6* deletion is consistent with previous evidence linking epithelial *STAT6* activation to spontaneous colonic inflammation [28]. In parallel, the effect associated with *Corylus avellana* extract was less consistent across the evaluated inflammatory markers, although a significant reduction in *NF-kB* expression was observed. This effect may be related to the bioactive and antioxidant constituents of the extract, which have previously been associated with reduced inflammatory signaling [26,29].

Persistent inflammatory stimuli may disrupt autophagy, impairing the clearance of intracellular components and pathogens, thereby contributing to epithelial barrier dysfunction and chronic intestinal inflammation [30]. In this study, genes involved in different stages of autophagy were upregulated in both experimental colitis and human UC. In patients with UC, increased expression of autophagy initiation genes was observed, including *ULK1*, *ULK2*, *BECN1*, *PIK3C3,* and *UVRAG* and *ATG9A*, which suggests transcriptional alterations in the autophagy initiation machinery [31,32,33,34]. Moreover, genes involved in phagophore elongation and autophagosome formation, including *ATG5*, *ATG10*, *ATG12*, *ATG16L1*, *ATG3*, *ATG7*, *MAP1LC3B,* and *SQSTM1,* were also upregulated [6,35,36]. Similarly, the experimental colitis mouse model showed increased *ATG16L1*, *MAP1LC3B,* and *SQSTM1* expression. Moreover, *STAT6* deletion significantly attenuated the expression of several of these genes, whereas *Corylus avellana* treatment showed a lower expression, although the differences were not statistically significant. These genes are involved in different aspects of the autophagic pathway, with *ATG16L1* being required for efficient LC3 lipidation and autophagosome biogenesis [37], while previous studies have used changes in the LC3-II/LC3-I ratio and p62 protein levels as indicators of altered autophagy in experimental colitis [38]. Alterations in *ATG16L1* expression impairs intracellular pathogen clearance, promoting the activation and persistence of intestinal inflammation and contributing to IBD pathogenesis [8]. These findings suggest that several autophagy-associated genes are transcriptionally altered in experimental colitis and human UC, which may indicate changes in the regulation of this pathway in the context of intestinal inflammation. However, additional studies should be performed, especially at the protein level, in order to further confirm the results obtained through gene expression analysis regarding the autophagic flux.

Autophagy and ERS are closely interconnected, as ERS can induce autophagy as a compensatory mechanism to restore cellular homeostasis, whereas impaired autophagy may further exacerbate unresolved ERS, establishing a self-amplifying cycle that contributes to intestinal inflammation [39]. Persistent ERS activates the UPR, an adaptive mechanism aimed at restoring ER homeostasis. However, sustained UPR activation may promote inflammatory and apoptotic signaling and contribute to epithelial dysfunction in UC [9,10]. In the present study, increased expression of *EIF2AK3*, *XBP1*, and *ATF6A* in experimental colitis indicated transcriptional alterations associated with the three major UPR-signaling pathways. A similar transcriptional pattern was also observed in human UC, where genes associated with the PERK (*EIF2AK3*, *ATF4*, *PPP1R15A*), IRE1 (*ERN1*, *XBP1*), and ATF6 (*ATF6*, *MBTPS1*) pathways were upregulated. These findings are consistent with previous evidence of Rodrigues et al., who demonstrated activation of the PERK/eiF2α and IRE1/XBP1 pathways in colonic mucosa from patients with UC and reported increased *EIF2AK3* expression [10]. Furthermore, increased expression of *CALR* and *HSPA5* observed in UC may reflect an adaptive response aimed at restoring protein-folding homeostasis, as these molecular chaperones can be induced by ATF6α [40]. Sustained ERS may amplify NF-kB-mediated inflammation and impair goblet cell and epithelial function, linking UPR signaling to inflammation and epithelial barrier dysfunction [9,41]. In experimental colitis, *STAT6* deletion reduced *EIF2AK3*, *XBP1,* and *ATF6A*, suggesting that it may attenuate UPR-associated transcriptional alterations. In contrast, *Corylus avellana* treatment did not induce a consistent modulation of the evaluated genes. Nevertheless, further functional analysis is required to determine whether these transcriptional alterations correspond to functional activation of the UPR pathways.

Apoptosis is closely interconnected with inflammation, autophagy, and ERS, and its excessive activation in intestinal epithelial cells may impair epithelial barrier integrity and contribute to IBD pathogenesis [42]. Accordingly, human UC samples showed upregulation of pro-apoptotic genes, including *BAX*, *BAD*, *BID,* and *BAK1*, together with increased *CASP3* expression. The disproportionate increase in *BAX* relative to *BCL2* further supports a pro-apoptotic phenotype [43], consistent with previous evidence linking inflammation and increased *BAX* expression to enhanced apoptosis in UC [44]. Overall, these findings suggest that enhanced apoptosis may represent a downstream consequence of persistent cellular stress and inflammation, further contributing to epithelial barrier dysfunction in UC.

The findings in the present study are limited by the absence of protein-level validation of the observed gene expression changes. The transcriptional analysis allowed the identification of several molecular alterations associated with experimental colitis and human UC, but changes in mRNA expression do not necessarily result in corresponding changes at the protein level. Therefore, future studies should include protein-level analyses to validate the identified molecular changes and provide a more comprehensive understanding of their biological relevance in UC.

Beyond transcriptional alterations, UC was associated with a marked miRNA dysregulation, suggesting the involvement of post-transcriptional mechanisms in intestinal inflammation. Among the selected miRNAs, miR-223-3p, miR-21-5p, and miR-155-5p were upregulated in both experimental colitis and human UC. Increased miR-155 contributes to intestinal immune activation, whereas its deficiency attenuates experimental colitis and Th1/Th17 responses while promoting M2 macrophage polarization [45,46]. Moreover, Chen et al. reported increased miR-155 expression and a positive correlation with TNF in experimental colitis [47]. miR-21-5p is also upregulated in intestinal inflammation and IBD and has been implicated in inflammatory responses and epithelial barrier dysfunction, reducing inflammatory cell infiltration and pro-inflammatory cytokine production [48]. Wu at al. similarly reported increased miR-21 expression in TNBS-induced colitis and reported model dependent differences in miR-21 and miR-223 expression [49]. Increased miR-223-3p expression in our study is consistent with previous evidence involving it in the crosstalk between inflammatory signaling and intestinal barrier dysfunction in TNBS-induced colitis [50]. Moreover, miR-223 can negatively regulate NLRP3 inflammasome activity, suggesting that its upregulation may also represent a compensatory mechanism limiting excessive inflammation [51]. Increased miR-146a-5p expression in UC may represent a regulatory response to persistent inflammation by negatively regulating inflammatory signaling through the IRAK1/TRAF6/NF-kB axis [52,53]. The concomitant upregulation of miR-21-5p, miR-155-5p, and miR-146a observed in our UC samples is consistent with the findings of Gunaltay et al., who reported increased expression of these miRNAs in UC colonic biopsies [54]. miR-146a-5p has also been linked to autophagy dysregulation in an in vitro model of colitis, where its inhibition promoted autophagy [55], while members of the miR-106 family may regulate autophagy through ATG16L1 [56]. On the other hand, reduced miR-29b-3p expression in UC could favor extracellular matrix remodeling, as the miR-29 family negatively regulates several extracellular matrix components and its downregulation has been associated with intestinal fibrosis [57,58]. miR-27b-3p is less characterized in UC, although studies have identified its implications in inflammatory signaling and autophagy regulation, including modulation of PPARα [59] and ATG10 [60], as well as its ability to modulate oxidative stress, apoptosis, and tissue remodeling [61,62]. Overall, similar miRNA expression changes were observed in murine experimental colitis and human UC biopsies, suggesting that these molecular alterations may be relevant to UC pathogenesis. The partial normalization following *STAT6* deletion, and to a lesser extent following *Corylus avellana* treatment, suggests that these interventions may modulate multiple components of the colitis-associated molecular response and is consistent with the changes observed at the gene-expression level.

miRNet analysis revealed a broad miRNA-target interaction network in which several of the selected miRNAs shared potential targets involved in inflammation (*IL6*, *TNF*, *NFKB1*), epithelial barrier integrity (*MUC2*, *OCLN*, *CLDN1*), autophagy (*ATG16L1*, *MAP1LC3B*, *SQSTM1*), and ERS (*EIF2AK3*, *XBP1*). However, these interactions were identified through bioinformatics analysis and were not experimentally validated in the present study. Therefore, the identified miRNA-target relationships should be regarded as potential regulatory interactions that require further investigation, including correlation analyses using matched expression data and functional validation to establish their biological relevance.

While previous studies have investigated individual molecular pathways or specific miRNAs in experimental colitis or human UC, the present study examines gene and miRNA expression profiling across multiple interconnected biological processes, including inflammation, epithelial barrier dysfunction, autophagy, and ERS. Moreover, the evaluation of *STAT6* deletion and *Corylus avellana* treatment in the TNBS-induced colitis model allowed their modulatory effects to be evaluated within the same molecular condition. The following evaluation of transcriptional and miRNA alterations in human UC mucosal colonic biopsies further enabled comparison between the experimental colitis and the human disease.

## 4. Materials and Methods

### 4.1. Mouse Models

All experimental procedures were performed in accordance with Directive 2010/63/EU and complied with both European and national regulations for the use of laboratory animals in scientific research. A total of 60 adult mice (1:1 sex ratio), comprising BALB/c wild-type and *STAT6*^−/−^ mice, were raised in the animal facility of the Western University “Vasile Goldiş” in Arad, Romania. Experimental colitis was induced by intrarectal administration of 2,4,6-trinitrobenzene sulfonic acid (TNBS) at a dose of 100 mg/kg body weight. A freshly prepared 5% (*w*/*v*) aqueous TNBS solution (Merck KGaA, Darmstadt, Germany) was mixed with absolute ethanol at a 1:1 (*v*/*v*) ratio, resulting in a final concentration of 2.5% TNBS in 50% ethanol. Food was withheld for 20 h prior to intrarectal administration of either physiological serum or TNBS. The *Corylus avellana* gemmotherapy extract used in this study was supplied by SC PlantExtrakt SRL Romania. The extract was obtained from fresh *Corylus avellana* buds using glycerin and 96% ethanol (1:1, *v*/*v*) as extraction solvent at a dry plant material-to-solvent ratio of 1:20. The preparation was subjected to cold maceration for 20 days with daily stirring, followed by decantation and pressing, as previously described by Balta et al. [15]. The phytochemical profile of the preparation was characterized by a high polyphenolic content, with flavonoids representing relevant bioactive constituents; hyperoside was identified as the predominant flavonoid, while chlorogenic acid was the major phenolic acid [15]. The extract was administered daily by oral gavage at a dose of 14.25 μL of extract/kg body weight, starting 48 h after TNBS or physiological serum administration and continuing for 14 days. The administered dose was selected based on a dosing protocol established during preliminary experimental testing. The animals were divided into six experimental groups (n = 10/group): group 1 (Control): physiological serum followed by water; group 2 (TNBS): TNBS followed by water; group 3 (*STAT6*^−/−^): physiological serum followed by water; group 4 (TNBS *STAT6*^−/−^): TNBS followed by water; group 5 (Corylus): physiological serum followed by *Corylus avellana* extract; group 6 (TNBS Corylus): TNBS followed by *Corylus avellana* extract. On day 15, all mice were sacrificed, and tissue samples of the colon and cecum were collected for analysis in sterile tubes containing RNAprotect Tissue Reagent (Qiagen, Hilden, Germany) and immediately stored at −80 °C until analysis.

#### 4.1.1. Quantitative PCR Gene Expression Evaluation and Data Analysis

Samples of intestinal tissue were homogenized with the Mixer Mill MM 200 (RETSCH, Haan, Germany), and total RNA was extracted using TRIzol Reagent (Thermo Fisher Scientific, Waltham, MA, USA), which was then tested for purity and integrity by Agilent Bioanalyzer 2100 (Agilent Technologies, Santa Clara, California, USA) and evaluated for quantity and quality with the NanoDrop 8000 Spectrophotometer (Thermo Fisher Scientific). Reverse transcription was performed using iScript cDNA Synthesis Kit (BioRad, Hercules, CA, USA), followed by quantitative Real-Time PCR (qPCR) performed on the ViiA7 Real-Time PCR System (Applied Biosystems, Foster City, CA, USA), using SYBR Select Master Mix 2X (Thermo Fisher Scientific), according to the manufacturer’s instructions. The housekeeping gene used for data normalization was glyceraldehyde-3-phosphate dehydrogenase (*GAPDH*), and its primer sequences can be found in Table 2, along with the primers for the selected genes. Relative changes in gene expression were determined using the 2^−ΔΔCt^ method and statistical analyses, and graphical representations were generated using GraphPad Prism version 9.0 (GraphPad Software Inc., San Diego, CA, USA). Differences among experimental groups were evaluated by one-way analysis of variance (ANOVA) followed by Bonferroni’s post-hoc multiple-comparison test. Data are presented as the mean ± standard deviation (SD), and differences were considered statistically significant at a *p*-value < 0.05.

#### 4.1.2. microRNA Expression Profile and Integrative Bioinformatic Analyses

miRNA expression profiling was carried out for the comparative evaluation of the expression level of 84 miRNA molecules with potential involvement in experimental colitis. After total RNA was extracted using TRizol Reagent (Thermo Fisher Scientific), reverse transcription was performed with the All-in-One miRNA First-Strand cDNA Synthesis Kit (GeneCopoeia, Rockville, MD, USA), followed by 1:10 dilution, according to the manufacturer’s recommendations. The miProfile Mouse Immunopathology miRNA qPCR Array plates (GeneCopoeia) were processed with the All-in-One qPCR Mix (GeneCopoeia), and amplification was performed on a ViiA 7 Real-Time PCR System (Applied Biosystems) according to the manufacturer’s instructions. qPCR data were analyzed using the Analysis Tool available from the manufacturer (GeneCopoeia; https://www.genecopoeia.com/product/gene-qpcr-array/#Analysis_Tool, accessed on 1 August 2026), based on the 2^−ΔΔCt^ method and expressed as fold change, while corresponding heatmaps were generated with the GeneGlobe Data Analysis Center (Qiagen; https://geneglobe.qiagen.com/us/analyze, accessed on 1 August 2026). For consistent interpretation of expression patterns, fold change values were subsequently converted to fold regulation values. For multiple-testing correction, the *p*-values obtained for the 84 miRNAs evaluated were adjusted using the Benjamini–Hochberg procedure to control the false discovery rate (FDR). FDR-adjusted *p*-values < 0.05 were considered statistically significant. miRNAs relevant to the biological processes investigated in the present study were subsequently selected for graphical representations using GraphPad Prism version 9.0 (GraphPad Software Inc.), and data are presented as mean ± SD. Functional target and network analysis were generated using miRNet 2.0 platform. For the global miRNA-target interaction network, experimentally validated interactions were obtained from miRTarBase v9.0 and TarBase v9.0, with no additional threshold applied. For the focused network representation, target genes associated with the biological processes investigated in the present study were selected from the global network. Functional enrichment analysis of the miRNA-associated target genes was performed using the Kyoto Encyclopedia of Genes and Genomes (KEGG) database. Among the significantly enriched pathways identified, six representative pathways were selected for presentation based on their relevance to the biological processes of interest.

### 4.2. Patients

The present study followed the ethical guidelines outlined in the 2013 Declaration of Helsinki. All participants included in the study signed a written informed consent form prior to biopsy sampling at Elias Emergency University Hospital. Samples were collected during colonoscopy procedures and consisted of a group of 14 patients with active UC (UC) and a group of 4 healthy individuals (Control). Colonic mucosal biopsies were collected in sterile tubes containing RNAprotect Tissue Reagent (Qiagen) and were frozen immediately at −20 °C.

#### 4.2.1. Total RNA Isolation and PCR Array Gene Expression Evaluation

Gene expression was comparatively evaluated between healthy individuals and patients with UC using RT^2^ Profiler PCR Array Human Arrays (Qiagen): the Autophagy array, comprising 84 autophagy-associated genes, and the Unfolded Protein Response array, comprising 84 UPR-associated genes. Total RNA was isolated from colonic mucosal biopsies using TRIzol Reagent (Thermo Fisher Scientific), and RNA integrity and concentration were assessed prior to cDNA synthesis. Reverse transcription was performed using the RT^2^ First Strand Kit (Qiagen), followed by qPCR on the ViiA 7 Real-Time PCR System (Applied Biosystems) using RT^2^ SYBR Green Mastermix (Qiagen), according to the manufacturer’s instructions. Data from each array were analyzed separately using the GeneGlobe Data Analysis Center (Qiagen; https://geneglobe.qiagen.com/us/analyze, accessed on 1 August 2026) to determine relative gene expression and fold regulation, as well as to generate heatmap clusters. KEGG pathway analysis was performed using ShinyGO version 0.86. The analysis was conducted for *Homo sapiens* to identify significantly enriched biological processes and pathways associated with the selected genes, including autophagy- and UPR-related pathways. The results were visualized using bar plots, with bar length representing fold enrichment and bar color indicating statistical significance based on the negative logarithm of the false discovery rate (−log10[FDR]). KEGG pathway maps were also generated to visualize the genes associated with the pathways of interest. For multiple-testing correction, the *p*-values were adjusted using the Benjamini–Hochberg false discovery rate (FDR), separately for the 84 autophagy-associated genes and the 84 UPR-associated genes, with *p*-value < 0.05 being considered statistically significant. Genes relevant to the biological processes investigated in the present study were subsequently selected for individual graphical representation using GraphPad Prism version 9.0 (GraphPad Software Inc.), and data are presented as mean ± SD.

#### 4.2.2. microRNA Expression Profile

The expression of selected mature miRNAs (miR-223-3p, miR-21-5p, miR-155-5p, miR-106a-5p, miR-146a-5p, miR-27b-3p, miR-29b-3p, miR-34a-5p) was evaluated in healthy individuals and patients with UC. After total RNA was isolated using TRIzol Reagent (Thermo Fisher Scientific), cDNA was synthesized with the All-in-One miRNA First-Strand cDNA Synthesis Kit (GeneCopoeia). qPCR amplification of miRNAs was performed using All-in-One miRNA qPCR Validated Primers (GeneCopoeia), Universal Adaptor PCR Primer (GeneCopoeia) and the All-in-One qPCR Mix (GeneCopoeia) on the ViiA 7 Real-Time PCR System (Applied Biosystems), according to manufacturer’s instructions. Statistical analyses and graphical representations were performed using GraphPad Prism version 9.0 (GraphPad Software Inc.), with the use of ANOVA algorithm and Bonferroni correction. Data are presented as mean ± SD, and *p* < 0.05 was considered statistically significant.

## 5. Conclusions

In the present study, comparative gene and miRNA expression profiling in experimental colitis and human UC revealed transcriptional alterations related to inflammation, epithelial barrier integrity, autophagy, ERS, and apoptosis, suggesting potential associations among these processes in UC pathogenesis. Integration of miRNA expression with target network analysis further identified potential associations across the investigated pathways, with miR-155, miR-223, and miR-146a sharing multiple potential target genes, while miR-27b-3p emerged as a candidate associated with several of the molecular processes. Both *STAT6* deletion and *Corylus avellana* treatment modulated colitis-associated molecular alterations, with *STAT6* deletion exerting a more pronounced effect at both gene and miRNA levels. Overall, these findings highlight *STAT6*-associated molecular alterations and selected miRNA-gene networks as relevant candidates for further functional investigation in UC.

## Figures and Tables

**Table 1:** Representative significantly enriched KEGG pathways associated with target genes of the selected dysregulated miRNAs.

KEGG Pathway	Hits	Adjusted *p*-Value	Target Genes
Regulation of autophagy	12	2.28 × 10^−17^	*ATG3*, *ATG5*, *ATG4B*, *ATG7*, *ATG12*, *ATG13*, *ATG10*, *ATG16L1*, *ULK1*, *ULK2*, *BECN1*, *IFNG*
Apoptosis	11	4.33 × 10^−12^	*BCL2*, *BCL2L1*, *BAD*, *BID*, *BAX*, *CASP3*, *CASP8*, *FAS*, *FADD*, *TNF*, *NFKB1*
TNF signaling pathway	9	4.03 × 10^−8^	*TNF*, *IL6*, *NFKB1*, *CASP3*, *CASP8*, *FAS*, *FADD*, *MMP9*, *ATF4*
Protein processing in ER	9	9.73 × 10^−7^	*EIF2AK3*, *XBP1*, *ERN1*, *ATF4*, *CANX*, *CALR*, *BCL2*, *BAX*, *BAK1*
Pathways in cancer	8	2.50 × 10^−4^	*CDKN2A*, *PTEN*, *MTOR*, *BCL2*, *BAK1*, *MMP9*, *NFKB1*, *CASP3*
Inflammatory bowel disease	4	7.59 × 10^−4^	*TNF*, *IL6*, *NFKB1*, *IFNG*

**Table 2:** Primer sequences for qPCR reaction.

Selected Genes	Forward Primer	Reverse Primer
*GAPDH*	GAGTCAACGGGGTCGT	TTGATTTTGGATCTCG
*MUC2*	GAAGCCAGATCCCGAAACCA	CCAGCTTGTGGGTGAGGTAG
*OCLN1*	GGCGGATATACAGACCCAAGAG	GATAATCATGAACCCCAGGACAAT
*CLDN1*	ACTCCTTGCTGAATCTGAACAGT	GGACACAAAGATTGCGATCAG
*MMP9*	GATGACGATGAGCTATGGACCTT	TCGGCGTTCCCATACTTCAC
*IL-6*	AGACAGCCACTCACCTCTTCAG	TTCTGCCAGTGCCTCTTTGCTG
*TNF-α*	CTCTTCTGCCTGCTGCACTTTG	ATGGGCTACAGGCTTGTCACTC
*NF-kB*	CCTGACTTTGAGGGACTGTATC	GGCTAGATGCAAGGCTGTT
*ATG16L1*	TGATGGTGCGTGGAATGATAG	CTTGATGGTTCCTCTGTTGTG
*MAP1LC3B*	AGATCCCAGTGATTATAGAGCGA	TTGCTGTCCCGAATGTCTCC
*SQSTM1*	CTGACACCCACTACCCCAGA	GTCCTTCCTGTGAGGGGTCTA
*EIF2AK3*	GCCTTTCTTGAACTCTCACC	CCGTGCTTTCTGTGAAGTGT
*XBP1*	GCTGAGTCCGCAGCAGGT	CAGGGTCCAACTTGTCCAGAAT
*ATF6A*	GTCTCATTTCGAAGGGATCAT	AGTCCTGCCCATTGATCACA

## Data Availability

The original contributions presented in this study are included in the article/Supplementary Material. Further inquiries can be directed to the corresponding author.

## Supplementary Materials

The following supporting information can be downloaded at: https://www.mdpi.com/article/10.3390/ijms27219411/s1.
