# Supplementary material for: Gene and microRNA Expression Profiling in Experimental Colitis and Human Ulcerative Colitis: Effects of STAT6 Knockout and Corylus avellana Treatment
Source: Int J Mol Sci. 2026 Oct 22;27(21):9411. doi: 10.3390/ijms27219411 (PMC13762818; doi:10.3390/ijms27219411)
Supplement: Supplementary file 1 [file ijms-27-09411-s001.zip › ijms-4561695-supplementary.pdf]

Table S1A. Layout of the miRNA qPCR array used for miRNA expression profiling. NC= negative control, HK= housekeeping miRNA, RT= reverse transcription control, PCR= positive PCR control.

	**1**	**2**	**3**	**4**	**5**	**6**	**7**	**8**	**9**	**10**	**11**	**12**
**A**	miR-125b-5p	miR-126-3p	miR-129-5p	miR-132-3p	miR-134-5p	miR-140-5p	miR-142-5p	miR-142-3p	miR-145-5p	miR-149-5p	miR-150-5p	miR-15a-5p
**B**	miR-15b-5p	miR-16-5p	miR-183-5p	miR-184-3p	miR-185-5p	miR-186-5p	miR-191-5p	miR-194-5p	miR-195-5p	miR-203-3p	miR-205-5p	miR-21-5p
**C**	miR-210-3p	miR-214-3p	miR-223-3p	miR-26a-5p	miR-26b-5p	miR-28-5p	miR-29b-3p	miR-30b-5p	miR-30c-5p	miR-335-5p	miR-34a-5p	miR-363-3p
**D**	miR-370-3p	miR-409-3p	miR-451	miR-574-3p	miR-9-5p	miR-99b-5p	miR-105	miR-155-5p	miR-182-5p	miR-298-5p	miR-299-3p	miR-325-3p
**E**	miR-383-5p	miR-31-5p	let-7d-5p	let-7g-5p	miR-130a-3p	miR-146a-5p	miR-146b-5p	miR-148a-3p	miR-18a-5p	miR-200a-3p	miR-20a-5p	miR-20b-5p
**F**	miR-34c-5p	miR-98-5p	miR-18b-5p	let-7a-5p	let-7c-5p	let-7e-5p	let-7f-5p	miR-135a-5p	miR-135b-5p	miR-181a-5p	miR-196a-5p	miR-19a-3p
**G**	miR-19b-3p	miR-23b-3p	miR-27a-3p	miR-27b-3p	miR-29c-3p	miR-30e-5p	miR-106a-5p	miR-299-5p	miR-21-3p	miR-9-3p	miR-138-1-3p	miR-31-3p
**H**	NC1	NC2	HK1	HK2	HK3	HK4	HK5	HK6	RT	RT	PCR	PCR

Table S1B. Fold regulation of miRNA expression in TNBS-induced colitis compared with Control mice.

miRNA	Fold Regulation	Adjusted *p* -value	miRNA	Fold Regulation	Adjusted *p* -value
miR-125b-5p	-3.23	6.00E-06	miR-105	2.19	2.20E-04
miR-126-3p	8.86	4.10E-05	miR-155-5p	3.17	8.00E-06
miR-129-5p	-2.41	1.00E-06	miR-182-5p	2	1.45E-04
miR-132-3p	-2.2	6.40E-05	miR-298-5p	-1.29	1.60E-03
miR-134-5p	-1.92	7.40E-05	miR-299-3p	-3.38	<1.00E-04
miR-140-5p	4.84	6.10E-05	miR-325-3p	-2.69	3.90E-05
miR-142-5p	-6.06	1.50E-05	miR-383-5p	-2.3	5.24E-04
miR-142-3p	1.04	3.59E-01	miR-31-5p	4.34	1.30E-05
miR-145-5p	-4.55	1.90E-05	let-7d-5p	-4.61	<1.00E-04
miR-149-5p	-3.71	1.00E-06	let-7g-5p	-3.6	6.00E-06
miR-150-5p	2.12	8.70E-05	miR-130a-3p	-2.63	3.70E-05
miR-15a-5p	3.53	4.00E-06	miR-146a-5p	4.03	3.50E-06
miR-15b-5p	2.07	8.15E-04	miR-146b-5p	2.01	9.00E-06
miR-16-5p	2	1.95E-04	miR-148a-3p	3.06	1.69E-04
miR-183-5p	4.11	4.20E-05	miR-18a-5p	1.36	2.15E-03
miR-184-3p	-3.95	8.60E-05	miR-200a-3p	-1.75	4.90E-05
miR-185-5p	-2.34	4.56E-04	miR-20a-5p	3.2	6.00E-06
miR-186-5p	-5.25	1.00E-05	miR-20b-5p	2.42	4.30E-04
miR-191-5p	3.43	2.50E-05	miR-34c-5p	-2.13	9.20E-05
miR-194-5p	-3.22	4.70E-05	miR-98-5p	3.47	1.00E-06
miR-195-5p	3.13	4.90E-05	miR-18b-5p	3.31	6.80E-05
miR-203-3p	7.13	2.00E-06	let-7a-5p	-4.47	<1.00E-04
miR-205-5p	3.56	1.50E-05	let-7c-5p	-2.66	2.00E-06
miR-21-5p	9.09	1.00E-06	let-7e-5p	-2.57	1.10E-05
miR-210-3p	2.84	2.00E-06	let-7f-5p	-3.8	5.00E-06
miR-214-3p	2.47	1.22E-04	miR-135a-5p	-4.22	2.30E-05
miR-223-3p	3.34	1.00E-06	miR-135b-5p	4.11	2.30E-04
miR-26a-5p	4.84	1.90E-05	miR-181a-5p	-3.18	<1.00E-04
miR-26b-5p	4.25	2.50E-05	miR-196a-5p	2.1	1.96E-04
miR-28-5p	3.4	3.80E-05	miR-19a-3p	5.43	1.40E-05
miR-29b-3p	-4.35	1.10E-05	miR-19b-3p	1.91	6.00E-06
miR-30b-5p	-2.87	1.06E-04	miR-23b-3p	1.25	7.21E-03
miR-30c-5p	5.05	2.00E-06	miR-27a-3p	2.11	3.00E-06
miR-335-5p	-2.21	7.60E-04	miR-27b-3p	2.24	7.10E-05
miR-34a-5p	-4.17	1.10E-06	miR-29c-3p	-3.48	3.55E-04
miR-363-3p	-3.16	<1.00E-04	miR-30e-5p	3.55	1.90E-05
miR-370-3p	-3.67	1.00E-06	miR-106a-5p	4.63	1.80E-05
miR-409-3p	-3.91	2.00E-06	miR-299-5p	-5.59	1.00E-06
miR-451	-4.25	9.20E-05	miR-21-3p	4.47	2.80E-05
miR-574-3p	-1.54	1.60E-04	miR-9-3p	-4.64	4.00E-06
miR-9-5p	5.22	1.20E-05	miR-138-1-3p	-3.09	4.80E-05
miR-99b-5p	4.21	3.40E-05	miR-31-3p	4.42	1.20E-05

Table S1C. Fold regulation of miRNA expression in TNBS-induced colitis treated with *Corylus avellana* extract compared with untreated experimental colitis.

miRNA	Fold Regulation	Adjusted *p* -value	miRNA	Fold Regulation	Adjusted *p* -value
miR-125b-5p	-2.69	3.15E-04	miR-105	1.76	2.05E-03
miR-126-3p	8.08	8.00E-03	miR-155-5p	2.43	1.23E-04
miR-129-5p	1.94	2.80E-04	miR-182-5p	-1.46	2.78E-04
miR-132-3p	-2.61	1.25E-04	miR-298-5p	-1.64	2.50E-02
miR-134-5p	2	1.58E-04	miR-299-3p	-2.21	2.40E-02
miR-140-5p	3.24	1.77E-04	miR-325-3p	-1.9	3.04E-04
miR-142-5p	-4.66	1.30E-02	miR-383-5p	-1.48	3.66E-04
miR-142-3p	3.12	3.00E-02	miR-31-5p	3.37	3.50E-03
miR-145-5p	-3.7	1.10E-02	let-7d-5p	2.51	1.80E-02
miR-149-5p	-1.53	8.26E-04	let-7g-5p	-2.06	2.82E-02
miR-150-5p	2.15	1.14E-03	miR-130a-3p	-2.13	1.30E-02
miR-15a-5p	3.47	2.76E-04	miR-146a-5p	3.28	1.40E-04
miR-15b-5p	1.8	7.80E-01	miR-146b-5p	1.63	3.14E-04
miR-16-5p	1.76	3.21E-04	miR-148a-3p	1.32	1.93E-03
miR-183-5p	3	5.00E-03	miR-18a-5p	-1.15	5.06E-02
miR-184-3p	-1.62	2.78E-04	miR-200a-3p	-1.66	5.70E-02
miR-185-5p	-2.21	5.84E-03	miR-20a-5p	2.9	1.46E-03
miR-186-5p	-2.36	3.50E-05	miR-20b-5p	1.76	4.69E-02
miR-191-5p	4.49	5.00E-02	miR-34c-5p	1.02	5.88E-01
miR-194-5p	-2.77	3.60E-05	miR-98-5p	1.68	1.05E-03
miR-195-5p	1.58	1.02E-01	miR-18b-5p	2.74	4.20E-02
miR-203-3p	7.3	2.60E-04	let-7a-5p	-3.3	4.10E-03
miR-205-5p	2.44	1.47E-04	let-7c-5p	-1.83	3.34E-03
miR-21-5p	8.06	4.10E-03	let-7e-5p	1.43	9.13E-03
miR-210-3p	2.66	3.80E-04	let-7f-5p	-3.02	1.80E-02
miR-214-3p	1.74	5.90E-03	miR-135a-5p	-1.87	2.89E-04

miR-223-3p	3.06	2.60E-01	miR-135b-5p	3.04	1.13E-04
miR-26a-5p	3.91	8.40E-01	miR-181a-5p	1.08	1.61E-01
miR-26b-5p	4.55	9.00E-01	miR-196a-5p	2.71	2.81E-04
miR-28-5p	1.73	7.60E-02	miR-19a-3p	4.19	2.50E-01
miR-29b-3p	-1.33	3.93E-01	miR-19b-3p	2.05	2.90E-01
miR-30b-5p	-1.94	1.52E-01	miR-23b-3p	-1.08	2.03E-01
miR-30c-5p	3.33	5.50E-01	miR-27a-3p	2.4	1.54E-04
miR-335-5p	1.01	8.58E-01	miR-27b-3p	1.9	1.40E-02
miR-34a-5p	-2.87	9.00E-04	miR-29c-3p	-3.12	1.06E-04
miR-363-3p	-1.77	2.41E-04	miR-30e-5p	3.3	4.00E-02
miR-370-3p	-2.1	9.10E-01	miR-106a-5p	4.32	8.00E-01
miR-409-3p	-1.76	5.00E-02	miR-299-5p	-3.1	4.00E-03
miR-451	-2.15	1.17E-04	miR-21-3p	4.25	2.00E-02
miR-574-3p	1.36	2.06E-03	miR-9-3p	-2.57	1.00E-02
miR-9-5p	3.96	4.60E-03	miR-138-1-3p	-2.9	3.60E-03
miR-99b-5p	2.81	1.40E-04	miR-31-3p	2.46	3.90E-02

Table S1D. Fold regulation of miRNA expression in TNBS-induced colitis in *STAT6*-/- mice compared with experimental colitis.

miRNA	Fold Regulation	Adjusted *p* -value	miRNA	Fold Regulation	Adjusted *p* -value
miR-125b-5p	-2.28	4.65E-04	miR-105	2	1.89E-04
miR-126-3p	6.92	1.60E-05	miR-155-5p	2.1	4.20E-05
miR-129-5p	1.42	5.39E-04	miR-182-5p	-1.61	2.52E-04
miR-132-3p	1.8	6.72E-04	miR-298-5p	-1.63	4.50E-05
miR-134-5p	-1.47	9.60E-05	miR-299-3p	-1.55	5.15E-04
miR-140-5p	2.39	5.00E-05	miR-325-3p	-1.57	1.27E-03
miR-142-5p	-3.55	1.50E-04	miR-383-5p	-1.55	6.10E-04
miR-142-3p	4.14	5.00E-04	miR-31-5p	3.97	1.24E-04
miR-145-5p	-2.42	3.10E-05	let-7d-5p	2.27	5.00E-04
miR-149-5p	-2.61	7.80E-05	let-7g-5p	-1.4	2.71E-03
miR-150-5p	1.43	1.62E-03	miR-130a-3p	-1.47	1.27E-04
miR-15a-5p	3.22	5.00E-04	miR-146a-5p	2.71	2.22E-05
miR-15b-5p	1.84	1.53E-04	miR-146b-5p	2.35	7.40E-05
miR-16-5p	1.74	1.18E-04	miR-148a-3p	1.44	1.81E-03
miR-183-5p	2.51	5.92E-04	miR-18a-5p	-1.03	3.78E-02
miR-184-3p	-1.46	9.70E-03	miR-200a-3p	-1.51	1.81E-03
miR-185-5p	-2.01	6.71E-04	miR-20a-5p	2.91	2.40E-05
miR-186-5p	-2.53	2.60E-05	miR-20b-5p	1.34	1.41E-04
miR-191-5p	3.54	5.00E-05	miR-34c-5p	-1.04	2.07E-01
miR-194-5p	-2.15	7.60E-05	miR-98-5p	2.32	1.12E-04
miR-195-5p	1.06	7.90E-02	miR-18b-5p	2.71	1.16E-04
miR-203-3p	7.58	1.00E-05	let-7a-5p	-3.4	3.80E-05
miR-205-5p	2.17	7.40E-05	let-7c-5p	-1.34	2.94E-03
miR-21-5p	7.06	3.00E-05	let-7e-5p	1.4	3.58E-03
miR-210-3p	2.13	7.70E-05	let-7f-5p	-1.77	1.22E-04
miR-214-3p	1.16	4.66E-02	miR-135a-5p	-2.11	1.61E-04
miR-223-3p	2.6	3.54E-04	miR-135b-5p	3.08	1.10E-05
miR-26a-5p	3.55	2.60E-05	miR-181a-5p	-1.79	3.76E-04
miR-26b-5p	2.59	3.48E-04	miR-196a-5p	2	3.00E-05
miR-28-5p	2.24	1.90E-05	miR-19a-3p	4.02	1.30E-05
miR-29b-3p	-1.48	6.82E-04	miR-19b-3p	2.28	1.05E-04
miR-30b-5p	-1.47	9.85E-04	miR-23b-3p	1.4	6.60E-03
miR-30c-5p	2.58	7.00E-04	miR-27a-3p	1.58	5.21E-04
miR-335-5p	1.02	7.24E-01	miR-27b-3p	1.85	1.12E-03
miR-34a-5p	-2.68	1.10E-05	miR-29c-3p	-3.11	9.40E-05
miR-363-3p	-3.05	2.00E-04	miR-30e-5p	3.81	1.60E-04
miR-370-3p	-2.31	4.00E-05	miR-106a-5p	2.81	7.30E-04
miR-409-3p	-1.8	5.00E-03	miR-299-5p	-3.15	8.00E-03
miR-451	-2.84	5.00E-05	miR-21-3p	2.85	2.04E-04
miR-574-3p	1.26	4.56E-03	miR-9-3p	-2.29	2.00E-03
miR-9-5p	3.1	1.00E-03	miR-138-1-3p	-2.55	3.70E-05
miR-99b-5p	2.01	5.20E-05	miR-31-3p	2.7	8.00E-02

Table S1E. Fold regulation of miRNA expression in TNBS-induced colitis in *Corylus avellana* treated mice compared with TNBS-induced colitis in *STAT6*-/- mice.

miRNA	Fold Regulation	Adjusted *p* -value	miRNA	Fold Regulation	Adjusted *p* -value
miR-125b-5p	1.18	1.47E-02	miR-105	1.14	1.73E-01
miR-126-3p	-1.17	4.98E-02	miR-155-5p	-1.16	5.33E-02
miR-129-5p	-1.37	8.87E-03	miR-182-5p	-1.1	1.27E-01
miR-132-3p	4.69	1.55E-03	miR-298-5p	1.01	9.02E-01
miR-134-5p	-2.94	1.55E-03	miR-299-3p	1.43	9.93E-03
miR-140-5p	-1.35	1.66E-02	miR-325-3p	1.21	3.75E-02
miR-142-5p	1.31	1.47E-02	miR-383-5p	-1.05	4.82E-01
miR-142-3p	1.33	2.47E-03	miR-31-5p	1.18	8.39E-02
miR-145-5p	1.53	2.47E-03	let-7d-5p	-1.11	4.14E-02
miR-149-5p	-1.7	1.55E-03	let-7g-5p	1.48	2.47E-03
miR-150-5p	-1.5	4.72E-03	miR-130a-3p	1.45	2.47E-03
miR-15a-5p	-1.08	3.30E-01	miR-146a-5p	-1.21	4.26E-02
miR-15b-5p	1.02	6.75E-01	miR-146b-5p	1.44	4.72E-03
miR-16-5p	-1.01	8.85E-01	miR-148a-3p	1.09	1.22E-01
miR-183-5p	-1.2	6.53E-02	miR-18a-5p	1.12	1.03E-01
miR-184-3p	1.11	8.38E-02	miR-200a-3p	1.1	8.38E-02
miR-185-5p	1.1	1.23E-01	miR-20a-5p	1	9.71E-01
miR-186-5p	-1.07	3.29E-02	miR-20b-5p	-1.32	1.06E-02
miR-191-5p	-1.27	1.55E-03	miR-34c-5p	-1.06	5.33E-02
miR-194-5p	1.28	1.47E-02	miR-98-5p	1.38	1.47E-02
miR-195-5p	-1.49	1.55E-03	miR-18b-5p	-1.01	8.85E-01
miR-203-3p	1.04	5.78E-01	let-7a-5p	-1.03	4.20E-02
miR-205-5p	-1.12	8.38E-02	let-7c-5p	1.37	1.55E-03
miR-21-5p	-1.14	8.79E-02	let-7e-5p	-1.02	7.94E-01
miR-210-3p	-1.25	4.16E-02	let-7f-5p	1.7	3.26E-03
miR-214-3p	-1.49	3.11E-03	miR-135a-5p	-1.13	9.00E-03
miR-223-3p	-1.17	1.35E-03	miR-135b-5p	1.01	8.85E-01
miR-26a-5p	-1.1	1.88E-01	miR-181a-5p	-1.92	2.47E-03
miR-26b-5p	-1.76	2,73E-03	miR-196a-5p	-1.36	1.96E-02
miR-28-5p	1.3	4.72E-03	miR-19a-3p	-1.04	4.82E-01
miR-29b-3p	-1.11	6.07E-02	miR-19b-3p	1.12	7.96E-02
miR-30b-5p	1.33	2.54E-03	miR-23b-3p	1.51	8.22E-03
miR-30c-5p	-1.29	1.43E-02	miR-27a-3p	-1.52	4.72E-03
miR-335-5p	1.01	8.85E-01	miR-27b-3p	-1.02	8.09E-01
miR-34a-5p	1.07	1.62E-02	miR-29c-3p	1	9.88E-01
miR-363-3p	-1.73	6.59E-03	miR-30e-5p	1.16	9.55E-02
miR-370-3p	-1.1	7.96E-02	miR-106a-5p	-1.54	3.11E-03
miR-409-3p	-1.03	4.26E-02	miR-299-5p	-1.02	8.53E-01
miR-451	-1.32	3.90E-03	miR-21-3p	-1.49	3.90E-03
miR-574-3p	-1.09	1.36E-01	miR-9-3p	1.12	5.18E-02
miR-9-5p	-1.28	1.47E-02	miR-138-1-3p	1.14	3.75E-02
miR-99b-5p	-1.4	3.23E-03	miR-31-3p	1.09	8.54E-02

Table S1F. Autophagy-related gene expression in UC compared with Control, assessed using the Human Autophagy RT² Profiler PCR Array.

Gene Symbol	Fold Regulation	Adjusted *p* -value	Gene Symbol	Fold Regulation	Adjusted *p* -value
AKT1	4.53	<1.00E-04	GABARAP	1.02	9.73E-01
AMBRA1	2.42	3.48E-05	GABARAPL1	2.67	2.42E-02
APP	3.44	6.22E-06	GABARAPL2	2.22	4.94E-03
ATG10	6.71	<1.00E-04	HDAC1	5.45	9.45E-04
ATG12	7.99	<1.00E-04	HDAC6	2.65	6.98E-03
ATG16L1	7.2	9.88E-06	HGS	-3.96	<1.00E-04
ATG16L2	1.86	1.25E-03	HSP90AA1	6.84	9.88E-06
ATG3	3.35	8.22E-04	HSPA8	3.03	1.17E-05
ATG4A	2.54	7.91E-02	HTT	5.68	3.23E-06
ATG4B	2.79	3.60E-05	IFNG	5.58	<1.00E-04
ATG4C	-1.18	2.01E-01	IGF1	4.16	2.74E-04
ATG4D	4.62	1.59E-05	INS	-2.3	7.13E-04
ATG5	9.32	<1.00E-04	IRGM	8.56	3.23E-06
ATG7	3.32	<1.00E-04	LAMP1	6.3	5.12E-04
ATG9A	8.41	<1.00E-04	MAP1LC3A	6.12	<1.00E-04
ATG9B	2.62	3.28E-02	MAP1LC3B	6.22	4.17E-05
BAD	3.98	9.88E-06	MAPK14	7.35	5.71E-05
BAK1	4.62	3.23E-06	MAPK8	7.83	5.92E-05
BAX	3.19	1.18E-04	MTOR	2.34	1.05E-03
BCL2	2.67	1.82E-04	NFKB1	5.97	<1.00E-04
BCL2L1	7.94	<1.00E-04	NPC1	1.97	1.17E-05
BECN1	2.95	8.40E-06	PIK3C3	4.52	8.40E-05
BID	5.77	<1.00E-04	PIK3CG	12.15	1.40E-03
BNIP3	3.44	3.48E-05	PIK3R4	9.61	1.18E-04
CASP3	4.36	3.23E-06	PRKAA1	2.28	3.66E-02
CASP8	3.83	<1.00E-04	PTEN	-4.98	3.23E-06
CDKN1B	2.18	9.88E-06	RAB24	2.1	8.16E-03
CDKN2A	13.63	9.80E-05	RB1	3.11	1.71E-04
CLN3	4.87	8.22E-05	RGS19	3.35	1.70E-04
CTSB	2.11	2.09E-02	RPS6KB1	4.53	2.21E-05
CTSD	-3.48	3.23E-06	SNCA	-2.54	8.40E-06
CTSS	5.19	1.71E-03	SQSTM1	7.33	<1.00E-04
CXCR4	5.23	<1.00E-04	TGFB1	1.45	1.14E-01
DAPK1	2.28	2.53E-02	TGM2	5.53	1.71E-04

DRAM1	-1.08	2.97E-01	TMEM74	-2.05	9.46E-03
DRAM2	3.15	8.40E-06	TNF	6.7	<1.00E-04
EIF2AK3	5.13	<1.00E-04	TNFSF10	5.62	5.02E-04
EIF4G1	-5.25	<1.00E-04	TP53	6.77	3.02E-05
ESR1	5.2	1.71E-03	ULK1	7.23	3.23E-06
FADD	4.17	3.71E-05	ULK2	6.08	<1.00E-04
FAS	5.42	1.46E-05	UVRAG	3.06	5.53E-05
GAA	3.82	2.66E-02	WIPI1	2.24	5.16E-04

Table S1G. Unfolded protein response-related gene expression in UC compared with Control, assessed using Human Unfolded Protein Response RT² Profiler PCR Array.

Gene Symbol	Fold Regulation	Adjusted *p* -value	Gene Symbol	Fold Regulation	Adjusted *p* -value
AMFR	-1.35	3.00E-02	OS9	2.08	2,82E-04
ATF4	3.32	3,06E-05	PDIA3	2.26	1.06E-02
ATF6	2.49	1,95E-04	PFDN5	2.43	4,41E-04
ATF6B	3.8	1,18E-04	PPIA	3.13	3.11E-03
ATXN3	4.68	<1,00E-04	PPP1R15A	3.04	2.65E-05
BAX	3.46	4.53E-05	PRKCSH	-1.34	1.62E-02
CALR	4.37	5,68E-05	RNF5	1.97	1.20E-03
CANX	3.27	1,33E-05	RPN1	-1.38	1.71E-02
CCT4	3.26	9,88E-04	SCAP	-3.95	<1,00E-04
CEBPB	7.1	2,39E-04	SEC62	3.28	1,33E-05
CREB3	2.64	3,10E-04	SEC63	3.24	6,46E-05
CREB3L3	2.58	1,30E-04	SEL1L	5.29	5,68E-05
DERL1	4.82	1,47E-04	SELENOS	2.43	4,03E-04
DNAJC10	-1.74	1,18E-04	SERP1	3.96	1,53E-05
DNAJC3	6.8	1,53E-05	SIL1	2.42	7,52E-04
EDEM1	6.41	<1,00E-04	SREBF1	-1.76	3,42E-05
EIF2A	7.4	5,68E-05	SYVN1	7.54	9,88E-06
EIF2AK3	4.86	3,36E-05	TCP1	3.18	1,42E-04
ERN1	5.15	3,42E-05	TOR1A	4.2	9,88E-06
ERN2	3.74	0.001921	UBE2G2	-1.5	6.44E-03
ERO1A	5.55	5,04E-05	UBXN4	2.87	5,51E-05
ERO1B	3.98	5,51E-05	UFD1	1.37	1.45E-01
ERP44	3.86	5,20E-04	UGGT1	2.67	5,68E-04
FBXO6	3.96	2,88E-04	USP14	5.68	6,00E-06
GANAB	2.01	1.92E-03	VCP	4.65	1,53E-05

GANC	3.53	6,00E-06	XBP1	5.67	1,18E-05
HSPA1B	3.29	3,76E-04	ADM2	4.95	5,97E-05
HSPA2	1.9	3,43E-04	ASNS	-2.46	<1,00E-04
HSPA4	2.41	9,80E-04	BEX2	2.76	7,13E-04
HSPA4L	3.62	4,63E-05	DDIT3	1.24	1.71E-02
HSPA5	4.07	5.76E-05	DNAJB9	1.79	2.04E-03
HSPH1	1.98	3,27E-04	GINS2	5.2	6,00E-06
HTRA2	-1.44	1.80E-02	HERPUD1	5.12	<1,00E-04
HTRA4	-4.33	<1,00E-04	INHBE	-1.42	1.74E-02
INSIG2	4.18	<1,00E-04	KCNMB3	23.5	2,19E-05
MANF	5.51	<1,00E-04	MCM4	3.39	1,19E-04
MAPK8	7.23	<1,00E-04	PCNA	2.76	1,82E-04
MAPK9	7.82	6,00E-06	RRM2	2.57	5.73E-03
MBTPS1	3.24	3.41E-02	SLC17A2	4.09	3,36E-05
MBTPS2	2.4	7.06E-03	TRIB3	2.41	5,60E-05
NPLOC4	6.76	1,40E-04	TYMS	4.62	2,45E-05
NUCB1	2.13	2,93E-04	UHRF1	4.86	4,63E-05
